# Multivariate genetic determinants of EEG oscillations in schizophrenia and psychotic bipolar disorder from the BSNIP study

**DOI:** 10.1038/tp.2015.76

**Published:** 2015-06-23

**Authors:** B Narayanan, P Soh, V D Calhoun, G Ruaño, M Kocherla, A Windemuth, B A Clementz, C A Tamminga, J A Sweeney, M S Keshavan, G D Pearlson

**Affiliations:** 1 Olin Neuropsychiatry Research Center, Hartford Hospital, Institute of Living, Hartford, CT, USA; 2 Department of Electrical and Computer Engineering, University of New Mexico, Albuquerque, NM, USA; 3 The Mind Research Network, Albuquerque, NM, USA; 4 Genetics Research Center, Hartford Hospital, Hartford, CT, USA; 5 Genomas Inc, Hartford, CT, USA; 6 Department of Psychology, University of Georgia, Athens, GA, USA; 7 Department of Psychiatry, University of Texas Southwestern Medical School, Dallas, TX, USA; 8 Department of Psychiatry, Beth Israel Deaconess Medical Center and Harvard Medical School, Boston, MA, USA; 9 Department of Psychiatry, Yale University School of Medicine, New Haven, CT, USA; 10 Department of Neurobiology, Yale University School of Medicine, New Haven, CT, USA

## Abstract

Schizophrenia (SZ) and psychotic bipolar disorder (PBP) are disabling psychiatric illnesses with complex and unclear etiologies. Electroencephalogram (EEG) oscillatory abnormalities in SZ and PBP probands are heritable and expressed in their relatives, but the neurobiology and genetic factors mediating these abnormalities in the psychosis dimension of either disorder are less explored. We examined the polygenic architecture of eyes-open resting state EEG frequency activity (intrinsic frequency) from 64 channels in 105 SZ, 145 PBP probands and 56 healthy controls (HCs) from the multisite BSNIP (Bipolar-Schizophrenia Network on Intermediate Phenotypes) study. One million single-nucleotide polymorphisms (SNPs) were derived from DNA. We assessed eight data-driven EEG frequency activity derived from group-independent component analysis (ICA) in conjunction with a reduced subset of 10 422 SNPs through novel multivariate association using parallel ICA (para-ICA). Genes contributing to the association were examined collectively using pathway analysis tools. Para-ICA extracted five frequency and nine SNP components, of which theta and delta activities were significantly correlated with two different gene components, comprising genes participating extensively in brain development, neurogenesis and synaptogenesis. Delta and theta abnormality was present in both SZ and PBP, while theta differed between the two disorders. Theta abnormalities were also mediated by gene clusters involved in glutamic acid pathways, cadherin and synaptic contact-based cell adhesion processes. Our data suggest plausible multifactorial genetic networks, including novel and several previously identified (DISC1) candidate risk genes, mediating low frequency delta and theta abnormalities in psychoses. The gene clusters were enriched for biological properties affecting neural circuitry and involved in brain function and/or development.

## Introduction

Schizophrenia (SZ) and psychotic bipolar disorder (PBP) are debilitating mental illnesses with complex genetic and epigenetic architectures.^1, 2^ Etiologies underlying SZ and PBP are poorly understood, however, these disorders at least partially share genetic features and biological factors.^3, 4^ Studying electrophysiological signatures in conjunction with genetic polymorphisms in both SZ and PBP may provide insight into similarities and differences in the underlying neurobiological mechanisms and facilitate the understanding of the functional alterations causing these disorders. Although a fraction of the risk for psychiatric illness is contributed by rare mutations in the form of copy-number variants, most risk variance is theoretically accounted for by a multifactorial genetic model^5, 6^ comprising multiple additive and interacting genes. In the multifactorial model, the cumulative effect of all the genes is large, while each gene contributes a small effect towards the overall biological risk. Genetic and clinical heterogeneity in psychiatric illnesses complicates the ability to detect and replicate genetic associations. A feasible approach to examine genetic sources in psychiatric disorders is to use intermediate phenotypes (also referred to as endophenotypes) that are heritable, state independent and hypothesized to be simpler in structure and in closer proximity to the source of biological susceptibility than the actual illness.^7^

The awake or eyes-open resting electroencephalogram (EEG) is an intrinsic, task-unstructured and oscillatory type of electrical brain activity quantified using amplitude or power of underlying frequency or spectral distribution. It is obtained in a straightforward manner and commonly used neurophysiological measure in psychiatric disorders with high heritability.^8, 9^ The primary source generating EEG oscillations is coherent neuronal firing, characterized by their frequency activity representing information processing.^10^ Various frequency ranges/bands are associated with different brain functions, in particular, they denote synchronization or temporal communication between brain regions; long-distance synchronization occurs at low frequencies, whereas short-range neuronal synchronization is evident at higher frequencies.^11^ Delta oscillations may reflect homeostatic and metabolic processes.^12^ Theta oscillations index learning, memory and cognitive performance.^13, 14^ Alpha oscillations constitute a major EEG phenotype, referred to as ‘default mode' or idling state, particularly with eyes closed. Alpha rhythms are functionally correlated with memory,^14^ attention allocation^15^ and default mode network activity.^16^ High frequency beta activity is associated with cortical excitability.^17^

Although the frequency composition of EEG during eyes-open and eyes-closed conditions differ, prior studies indicate abnormal EEG oscillatory activity in SZ and PBP during both states.^18^ Recently, a multisite study demonstrated shared low frequency EEG abnormality in SZ and PBP probands with moderate heritability (~0.2–0.3) and relative risk for their first-degree relatives.^19^ Increased eyes-open^18, 19, 20, 21^ delta activity in SZ reflects frontal lobe pathology. Abnormal theta oscillations are present in both SZ and PBP and may index a general psychosis biomarker.^20^ Theta-alpha^22^ and high frequency beta^20, 23^ deviations during eyes-closed condition are associated with biological vulnerability to SZ, with abnormal gamma activity occurring among SZ and bipolar disorder probands and their relatives.^20^ Genetic studies of EEG frequency activity exist in the literature, but only one SZ report found a significant association of COMT genotype with low frequency (delta and theta) activity.^20^ Thus, there is scarcity of studies investigating genetic attributes, in particular polygenic factors mediating EEG oscillatory abnormalities in SZ and PBP, despite their possibly critical importance for understanding the functional correlates of the psychotic process. Various studies have examined the genetic underpinnings of EEG oscillations in other psychiatric studies using univariate approaches. Alpha activity was associated with COMT polymorphism in females with anxiety disorders^24^ and with an exon variant of GABA_B receptor gene.^25^ Family-based pedigree studies identified significant association between alpha activity and CRH-BP variants,^26^ theta activity and SGIP1 variants^27^ and a linkage between rs279836 single-nucleotide polymorphism (SNP) in the GABARA2 receptor gene and mid-beta frequency activity.^28^

Most commonly used genetic association analyses methods include: (i) genome-wide association including linkage analysis or (ii) the candidate gene approach. Both genome-wide association and candidate gene approaches treat SNPs independently in association with a unitary phenotype using univariate analyses, while linkage studies use pedigree-based family structure to locate a broad region across the genome where the influence of the phenotype is localized. The genome-wide association approach does not require prior hypotheses on the gene function but the candidate gene approach uses *a priori* knowledge of biological processes influencing an illness. Both approaches disregard the plausible simultaneous coupling between SNPs based on a multifactorial gene model and the former is limited by requiring very large sample sizes to account for the necessary multiple comparison corrections. These limitations can be minimized by using a statistically efficient data-driven multivariate association approach based on parallel independent component analysis (para-ICA)^29^ suited for modest-sized samples. This novel technique examines the simultaneous association between linearly combined SNP variants (based on additive model) treated as a single entity to linearly coupled phenotypes from a comprehensive set of biomarkers. Recent studies suggest a complex genetic effect subserving the differential mechanisms underlying the psychosis etiology. The para-ICA method is well suited for examining the polygenic architecture mediating EEG frequency abnormalities in psychoses and has been previously used in imaging, behavioral and EEG studies.^30, 31, 32, 33^

In this study, we sought to accomplish the following: (1) determine multifactorial candidate risk genes influencing EEG frequency activity in SZ and PBP, (2) determine whether the genetic polymorphisms regulate EEG frequency abnormalities in SZ or PBP or both disorders and (3) examine the biological pathways associated with the pathophysiology of SZ and PBP. The primary hypothesis we propose in this study is that the multivariate para-ICA approach would reveal novel and/or previously identified candidate risk genes and pathways including neurotransmitter signaling, synaptic networks and neurodevelopmental mechanisms associated with psychosis in SZ and PBP.

## Materials and methods

### Bipolar-schizophrenia network on intermediate phenotypes study

The genotype–phenotype multivariate association analysis was conducted using para-ICA of SNP and EEG frequency activity from SZ, PBP probands and healthy controls (HCs) from the BSNIP (Bipolar-Schizophrenia Network on Intermediate Phenotypes) study,^34^ a multisite (six sites; Baltimore, Boston/Detroit, Chicago, Dallas, Hartford) collaborative project (www.b-snip.org) aimed at characterizing intermediate phenotypes across psychosis and identifying their genetic determinants.

### Participants

The study sample consisted of 306 subjects organized into three groups including 105 SZ, 145 PBP probands and 56 HCs. The sample was selected such that the subjects had both eyes-open EEG data (a subset of *n*=1091; Narayanan *et al.*^19^) and genetic data (*n*=620 subjects were genotyped), both being subset of ~2500-person BSNIP cohort^34^ (see Supplementary Material). SZ and PBP patient groups were not matched on age, sex or ethnicity with HC (see Table 1). Medication details for probands are listed in Supplementary Table S1. Probands met the SCID DSM IV-TR^35^ criteria for SZ or BP I disorder with psychosis (as defined in ref. 36) and those with schizoaffective disorder depressive and mania subtype diagnoses were assigned to SZ or PBP groups, respectively, as previously documented.^37^ HCs comprised subjects with no DSM Axis 1 disorder and no first-degree relative with a psychotic illness. Exclusion criteria for all subjects included known central neurological illness, substance abuse (within 6 months) or dependence within 2 years, positive pregnancy or positive urine toxicology screens. Participants gave institutional review board approved written informed consent at the respective sites after a detailed explanation of the study.

### Eyes-open EEG data acquisition

EEG recordings were acquired with identical 64-channel (Ag/AgCl sensors; impedance was kept <5 KΩ) Neuroscan equipment (Quik-Cap, Compumedrics, El Paso, TX, USA), while the subjects were in eyes-open state (see Supplementary Material). The data were obtained by trained technicians at all the sites. To maintain consistency, recording equipment was adjusted to identical specifications at all the sites. Electrodes were positioned per the standard 10-10 system with forehead as ground and nose as reference. Reference electrooculogram recordings were collected by placing one electrode at the inner and another at the outer canthus of the left and right eye. Subjects were instructed to sit quietly on a straight-backed chair in a shielded booth with eyes open focused on a fixation cross on a monitor for 5 min. During acquisition, EEG data were digitized at sampling rate of 1000 Hz and band pass filtered between 0.5 and 100 Hz.

### SNP data collection

DNA was extracted using blood samples collected from participants. Approximately one million SNP variants (1 140 419) across the whole genome were obtained by genotyping with Illumina Human Omni1-Quad chip and BeadArray platform at Genomas, Hartford Hospital.

### EEG data processing

Raw EEG data were processed and artifact rejected (see Narayanan *et al.*^19^ and Supplementary Material for details) using EEGLAB.^38^ Data were resampled to 250 Hz and filtered between 0.5 and 50 Hz. The accepted epochs after quality control were visually inspected by trained research personnel to ensure that the data quality was not compromised and no major data cleaning was carried out in this step.

### Frequency transformation and data reduction using group-independent component analysis

Clean epochs were converted to the frequency domain using Fourier transform with a Hamming window. Frequency amplitude was obtained by taking square root of frequency-power to form the instantaneous amplitude profile of each trial. Frequency data <1.5 Hz were excluded from further analysis to safeguard from slow lateral eye movements. We compressed EEG frequency-transformed data with the group-independent component analysis approach (see Supplementary Figure S1 and Supplementary Material) used in prior imaging and EEG studies^39, 40^ using the GIFT toolbox (GIFT v1.3c; http://icatb.sourceforge.net).^41^ The EEG frequency data in this study were a subset of the primary group-independent component analysis described in Narayanan *et al.*^19^ The mean number of epochs for each group is listed in Table 1.

### SNP data processing

**Figure 1:**
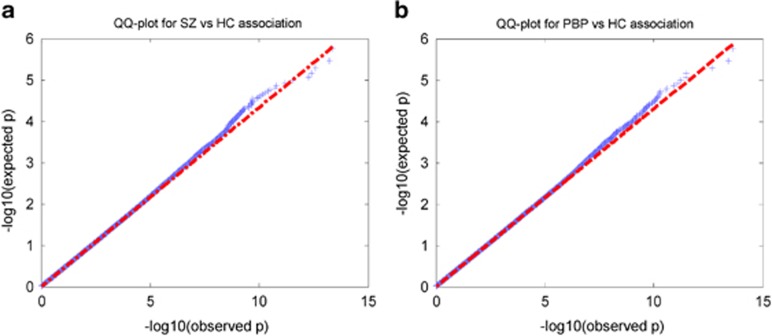
Q–Q plot of theoretical and empirical *P*-values from logistic regression for (**a**) schizophrenia (SZ) vs healthy controls (HCs) and (**b**) psychotic bipolar disorder (PBP) vs HCs. Logistic regression was applied to individual markers in case–control fashion for both SZ and PBP groups.

Raw SNP data in categorical format (homozygous (AA, BB) and heterozygous (AB or BA)) were numerically coded for the number of minor alleles (AA=0, AB=1 and BB=2, assuming B is a minor allele) based on additive model. Supplementary Figure S1 illustrates the processing pipeline for quality control of SNPs. The first stage of SNP data processing removed individuals with poor genotyping quality by inspecting for discordant sex, exceeding missing rate (>3%), abnormal heterozygosity (>3 s.d. from mean) and unusual relatedness between individuals (identity by descent >0.1875). Exclusion criteria for individual SNPs^42^ were as follows: minor allele frequency <5% call rate <98% *P*<0.00001 for deviation from Hardy–Weinberg expectation (in unrelated unaffected individuals); linkage disequilibrium >0.8 in block sizes of 10 kb; significantly differing genotype call rates between cases (SZ and PBP probands) and controls (*P*<0.00001). There were 575 687 autosomal SNPs after quality control analysis. SNP data were adjusted for hidden population stratification (PCA-based eigenstrat) to minimize false positives by identifying and correcting those PCA components (top three in the current sample)^43^ for which the loading coefficients (LCs) were significantly associated with the self-reported ethnicity. No significant difference in LCs was detected between cases and controls. The q–q plot (see Figure 1) shows no substantial inflation in the SNP data. To gain statistical power, the SNP quality control and univariate analyses were carried out on all 620 subjects with genotype data, but only data specific to subjects (*n*=306) from the current sample were selected for multivariate association analysis.

One challenge with para-ICA is the weak aggregate signal quality obtained from the linear combination of numerous SNPs. To combat this issue, we carried out a univariate analysis based on logistic regression between cases and controls at each SNP, a strategy employed in prior studies.^31, 32^ The regression was applied separately for each patient group (SZ, PBP) vs controls. SNPs with uncorrected significance level *P*<0.025 in either of the two univariate analyses were merged and then queried using online databases dbsnp (http://www.ncbi.nlm.nih.gov/SNP/) and genome variation server (http://gvs.gs.washington.edu/GVS137/) to determine the functional annotation for each marker. None of the SNPs exceeded the genome-wide significance level. A total of 10 422 SNPs with gene annotation were selected at uncorrected *P*<0.025 from the regression analyses for the association analysis. Although the *P*-value cutoff is arbitrary, we chose a threshold of 0.025 to ensure an optimum sample size to SNPs ratio^44^ for reliable operation of the multivariate association.

### SNP-EEG para-ICA association

The SNP and spatio-spectral EEG data were concurrently assessed using the para-ICA algorithm.^29, 30^ ICA is a data-driven multivariate tool that uses higher-order statistics for separating maximally independent sources from linear mixture, based on presumed source independence, to provide better signal-to-noise ratio by identifying and eliminating unstructured noise sources from the data.^45^ Para-ICA is a modified ICA technique applied to two modalities by including an additional objective function of maximizing the linkage between the two feature sets apart from separately maximizing the source independence on each based on information theory.^30^ Para-ICA simultaneously evaluates independent SNP and frequency components and dynamically computes the linkage between the two data domains to update the components to optimize the bi-modal association (see Supplementary Material for advantages). A genotype and phenotype data matrix was constructed from SNP (306 × 10 422) and frequency-based spatial weights (306 × 512), respectively. The two pooled data (SZ+PBP+HC subjects) matrices were jointly processed by para-ICA (Supplementary Figure S2). Model order for spatial oscillatory EEG weights was estimated to be 5 based on minimum descriptor length criteria,^46^ a common strategy used in prior studies. For the SNP data, the number of independent components was chosen as 9 based on the consistency tool that checked for the maximum reliability of the components.^47^ The accuracy of the para-ICA correlation and the component stability was tested using a leave-one-out cross-validation test with same parameters used as in the original run. The EEG and SNP component in the each significantly associated pair from the original run was correlated with components from each run of the leave-one-out analysis (*n*=306 runs). The component in each modality in each run that best matched the original pair was identified based on correlation. The average within modality correlation from different runs for each significantly associated pair was used as the final reliability index.

### Code availability

Para-ICA is implemented in the fusion ICA toolbox V2.0c (http://icatb.sourceforge.net) in Matlab (The Mathworks, Natick, MA, USA) platform.

### Statistical analysis

The multivariate genotype–phenotype association was assessed by computing Pearson's correlation between the SNP and EEG LCs. The current sample was not evenly distributed across age, sex, race and data collection site; hence their effects were controlled for via partial correlation by including them as factors and adjusting the significance levels (*P*-values) of the association. Such a strategy has been used in prior studies to account for effects of confounding factors.^30, 32^ Further, the significance levels were corrected for multiple comparisons (*P*=0.05/45) across all component combinations (5 × 9=45). The major SNPs and EEG frequency activity contributing to the association were chosen by setting a threshold of |Z|⩾2 for both modalities. LCs were assessed for group difference using two-sided t-test after testing for normality. Pearson correlation with chlorpromazine equivalents (see Table 1), PANSS positive, negative, general scores and schizo–bipolar scale were also evaluated.

### Enrichment analysis

GeneGo software from Metacore (Thompson Reuters, New York, NY, USA) was used to identify canonical pathways enriched by functionally related candidate genes influencing EEG frequency activity. The process networks, biological and metabolic properties associated with the gene clusters were determined based on GeneGo's proprietary database. Enrichment was measured by the degree of overrepresentation of functionally tied gene cluster in *a priori* known pathways and network processes. Statistical significance associated with enrichment was computed based on hypergeometric distribution: *P*-values were false discovery rate corrected for multiple comparisons.

## Results

### Eyes-open EEG frequency components from group-independent component analysis

The eight oscillatory components derived from group-independent component analysis comprised two delta, one theta, one slow alpha, two fast alpha, one slow beta and one fast beta activity, with a noticeable peak within the respective frequency ranges that characterize EEG frequency bands (see Supplementary Figure S3). Scalp topography weights are emphasized (correlated) or de-emphasized (anti-correlated) with respect to the peak of mean frequency component curve and represent the strength of connection between each lead and the associated frequency component. The description of the frequency components are described elsewhere^19^ (also refer to Supplementary Material).

### Multivariate association of gene and EEG oscillations

**Figure 2:**
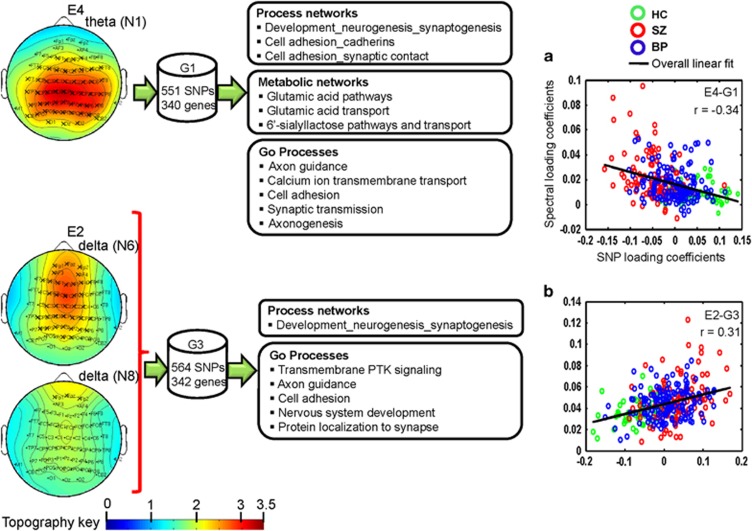
Spatial maps of eyes-open EEG frequency activity and associated genetic networks derived from parallel independent component analysis. (**a**) E4-G1 component pair, (**b**) E2-G3 component pair. E4 comprised posterior theta activity. E2 comprised two anterior delta activities. The spatial weights were converted to *Z*-scores. ‘X' indicates those electrodes for which the weights exceeded threshold |Z|=2. Biological pathways and process networks associated with each gene network from enrichment analysis are also displayed. The scatter plot of loading coefficients for the frequency-specific spatial components and genetic networks is also shown. BP, bipolar disorder; EEG, electroencephalogram; HC, healthy control; SNP, single-nucleotide polymorphism; SZ, schizophrenia.

**Figure 3:**
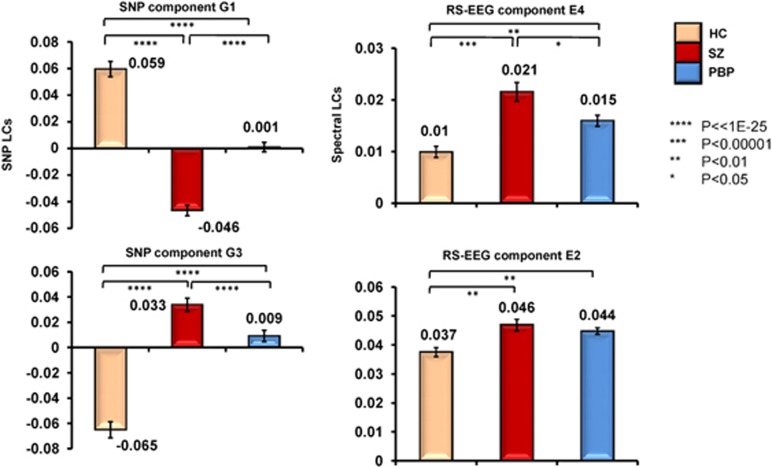
Mean loading coefficients (LC) for spatio-spectral EEG components and gene networks for schizophrenia (SZ) (*n=*105), psychotic bipolar probands (PBP; *n*=145) and healthy controls (HC; *n*=56). EEG LC represents each subject's contribution to the spatial weights associated with the delta and theta frequency components. Genetic LC represents each subject's contribution to the genetic network. E4 is posterior theta activity. E2 included two anterior delta components. Error bars represent standard deviation. *Post hoc* comparisons included pairwise *t*-tests between HCs and SZ and PBP probands. EEG, electroencephalogram; SNP, single-nucleotide polymorphism.

Para-ICA identified five spatio-spectral EEG and nine SNP components from the pooled data (SZ+PBP+HC). EEG components E4 and E2 were significantly associated with genetic components G1 (*r*=−0.34, *P*<4.4E−09) and G3 (*r*=0.31, *P*<1.1E−7), respectively (see Figure 2). The prominent EEG frequency oscillations in components E4 and E2 were posterior theta and anterior delta activity, respectively. The genetic component G1 comprised 551 SNPs/340 unique genes, while G3 included 564 SNPs/342 distinct genes. The top 20 most significant genes from G1 and G3, their *Z*-scores and associated functions are listed in Table 2. LCs were normally distributed and between-group variance was statistically equivalent. *Post hoc* analyses revealed significant group differences in LC of E2 (delta activity), E4 (theta) and both genetic networks between HCs and both probands. Theta activity and the LC (representing minor allele frequency) of two genetic networks differed between the SZ and PBP probands (see Figure 3). Theta activity was positively associated with schizo–bipolar scale (*r*=0.17, *P*<0.009) scores. No significant correlation was observed with PANSS scores. Both E4 and E2 were not significantly correlated (*r*=0.13, *P*=0.15 and *r*=−0.06, *P*=0.47) with chlorpromazine equivalents. Reliability test by leave-one-out cross-validation revealed an average within modality correlation >0.9 for both EEG and SNP component, indicating stable structure across several runs of the para-ICA.

### Enrichment analysis

Major process, metabolic networks and gene ontology processes enriched in G1 (see Supplementary Table S2) were as follows; process networks: development neurogenesis (synaptogenesis) and cell adhesion (cadherins, synaptic contact); metabolic networks: glutamic acid pathways and transport; gene ontology processes: including (but not limited to) axon guidance, calcium ion transport, cell adhesion and synaptic transmission. No major pathway maps associated with G1 were significant. Development neurogenesis (synaptogenesis) was the primary process network associated with G3. Prominent gene ontology processes enriched in G3 included transmembrane receptor protein tyrosine kinase signaling, axon guidance, cell adhesion and nervous system development. None of the pathways and metabolic networks was significantly enriched in G3 after false discovery rate correction. Brain expression scores derived from Allen brain database (www.brain-map.org) for the top 20 genes and related pathways are given in Supplementary Table S3.

## Discussion

EEG oscillations characterize intrinsic brain activity associated with cortical information processing and dynamic integration within and between brain regions. Abnormal low and high frequency activity are present in SZ and PBP with slow wave abnormalities including delta, theta and alpha common to both disorders. EEG activity exhibits heritable (~0.2–0.4 in BSNIP sample^19^) characteristics in family-based studies reflecting moderate genetic control over EEG frequency activity. Our estimates were lower compared with pedigree-based analyses, likely due to less dense kinship structure in our sample (most probands were represented by one relative). With such lower estimates compared with heritability of the illness, the utility of intermediate phenotypes for gene mapping is open for criticism, but the simple genetic architecture of quantitative biological measures offers powerful gene characterization of psychiatric illnesses.^48^ Etiological pathways associated with SZ and PBP are elusive and biological mechanisms underlying EEG frequency activity in both disorders are also undetermined. Thus, as a preliminary step, we examined a multi-loci genetic model using para-ICA that jointly identifies both synergistic genes and associated EEG frequency activity in SZ and PBP by relating the underlying hidden structures from both modalities. EEG frequency components were generated by filtering spectral data into data-driven bands using ICA as opposed to the traditional filtering-based frequency analysis.

### Multivariate gene-EEG oscillatory association

The most significant association pair was G1-E4. Gene network G1 was negatively correlated with E4; indicating decreased (increased) linear combination of MAF variations from gene clusters in G1 was associated with increased (decreased) theta activity. Similarly, the G3-E2 network pair was positively associated, reflecting increased MAFs from genes in G3 related to increased delta activity.

### Delta and theta activity

EEG frequency components E2 (delta) and E4 (theta) demonstrated increased loading scores in SZ and PBP compared with controls. The current finding of increased anterior delta and posterior theta activity in both SZ and PBP is consistent with prior magnetoencephalography^49^ and EEG studies.^18, 20, 21^ Augmented delta oscillations in both probands may index metabolic frontal lobe dysfunction.^12^ Increased theta activity was localized to the central and posterior regions in both proband groups with more severity in SZ compared with PBP. Cortical–hippocampal circuits are key generators of theta oscillations that have a crucial role in memory-encoding process,^14^ spatial information processing^50^ and regulating synaptic plasticity.^51^ Hippocampal cell discharges are a common phenomenon noted in all psychoses and provide a plausible neurophysiological model for psychosis-related disorganization.^52^ Thus, abnormal low frequency delta and theta oscillations might have a key role in psychoses common to SZ and PBP. Venables^20^ reported an association between low frequency activity and COMT gene variants.

### Gene networks

Para-ICA-based multivariate association jointly identifies both synergistic genes and associated phenotypes by relating the underlying structural patterns from both modalities. The main advantages of para-ICA are the statistical efficiency from small sample size achieved by combining high-dimensional SNP data, rather than accounting for each SNP through multiple comparison correction and data-driven approach. Each individual gene in the cluster is associated with a weight reflecting its contribution to the overall genotype–phenotype linkage. We first describe the brain-related functionality of the top dominant genes ranked by *Z*-scores and then the major biological pathways and processes of functionally combined gene groups mediating EEG oscillatory abnormality in SZ and PBP probands.

### Dominant genes with multiple SNP hits

#### Gene network (G1) mediating theta abnormality

The top-ranked gene was MSRA (including two SNPs), a reductase enzyme that converts methionine sulfoxide to methionine and serves as an antioxidant to guard against protein oxidative damage. Two prior reports identified MSRA as a SZ candidate risk gene.^53, 54^ Reduced antioxidant defense system causes an imbalance in free radicals such as proteins and lipids seen in SZ.^55^ Oxidative stress induces abnormal neuronal processes implicated in pathogenesis of psychosis in neuropsychiatric diseases,^56^ reflected by the lower total antioxidant measurement in both SZ and PBP compared with controls. Other highly ranked genes were CD200 that encodes for a type-1 membrane glycoprotein, expressed on neurons, involved in microglial activation and has a pivotal role in immune system, a known pathway associated with neuropathology of SZ.^57^ CLTCL1 belongs to the clathrin chain family and encodes a protein of polyhedral-coated synaptic vesicles that are recycled in nerve terminals facilitating unhindered neuronal synaptic transmission. CYP2C19 encodes the cytochrome P450 2C19 enzyme that is involved in the metabolism of psychoactive drugs and antidepressants including selective serotonin reuptake inhibitors. The expression of this gene in mouse models is related to decreased hippocampal volume and altered dentate gyrus neuronal density.^58^ Multiple SNPs were identified within DISC1, a well-known SZ and PBP risk gene. Some of the primary functions of DISC1 include cell proliferation, neutrite outgrowth, neuronal migration, cortical development, axonal guidance, synapse formation and adult neurogenesis. DISC1 is associated with P300 amplitude,^59, 60^ another phenotype of neuronal reactivity affected in both SZ and PBP.^61, 62, 63, 64^ Structural brain changes including reduced hippocampal volume, reduced prefrontal cortical gray matter and enlarged lateral ventricles are commonly associated with psychosis in both disorders and are controlled by DISC1.^65^ Moreover, this gene regulates synaptic function at glutamatergic synapses and its expression is associated with glutamate release.^66^ Preliminary evidence suggests that DISC1 contributes to neuronal migration and adult neurogenesis within the hippocampus.^67, 68^

#### Gene network (G3) mediating delta abnormality

The most significant and frequently identified gene in G3 was CACNA1I, a calcium channel, voltage-dependent, T type, alpha 1i subunit (CA_v_3.3) protein. Ligand and voltage-gated calcium channels alter neuronal excitability by controlling the entry of calcium ions into excitable cells, modulate calcium signaling and neurotransmitter release and regulate neuronal firing, a critical aspect of brain-related information processing.^69^ Similar voltage-gated calcium channel-related genes (CACNA1C and CACNB2) are common risk markers for several neuropsychiatric disorders.^4, 70^ Another gene with multiple SNP hits was NTRK3, a neurotrophic tyrosine receptor kinase protein linked to risk for PBP,^71^ autism^72^ and mood disorders.^73, 74^ A prior study reported a possible association with SZ via hippocampal dysfunction.^75^ NTRK3 gene variants are also associated with white matter integrity in brain regions impaired in neuropsychiatric disorders.^76^ Deficits in NTRK3 receptors cause diminished hippocampal axonal arbotization and synaptic densities.^77^ Further, these proteins are major elements in neuronal survival,^78^ axonal growth^79^ and synaptic plasticity.^80^

### Process and networks

A main objective of the present study was to identify gene clusters/networks in functionally related biological processes regulating EEG frequency abnormalities in psychoses. Pathway analytic approaches and gene network ontologies are still an active field of development, but existing techniques use numerous gene annotations databases with varying statistics, thus requiring improvement of existing annotations.^81^ However, it is important to use such techniques to establish key networks mediating complex psychiatric disorders to gain insight into mechanistic and biological roots associated with these diseases. As derived from pathway analyses in GeneGo, the most prominent process network mediating delta and theta abnormalities in psychoses was developmental neurogenesis (synaptogenesis). In addition, cell adhesion processes (through cadherins and synaptic contact) were involved in controlling theta abnormalities. Neurogenesis and synaptogenesis are among critical processes in brain development and function that might have a causal role in psychiatric disorders.^82, 83^ In particular, hippocampal neurogenesis is associated with learning, memory and synaptic plasticity.^84^ Abnormal hippocampal neurogenesis is associated with depression and several psychiatric disorders.^85^ In the present study, several genes including DLGAP1, NRXN3, ERBB4, DLG2, WNT3, APBA1, NRG3, ERC2, PARK2, LARGE and ACTG2 from G1 were involved in neurogenesis-related processes. Among these are several previously identified SZ and PBP candidate risk genes. Animal models of SZ indicate impaired adult neurogenesis that is normalized following treatment.^86, 87^ Cadherins are a group of glycoproteins participating in Ca^2+^-dependent cell–cell adhesion process engaged in adherens junction formation for cell binding. Cadherins and protocadherins have a crucial role in several nervous system functions including neural tube regionalization, neuronal migration, controlling neural circuitry, synapse formation, maintenance and brain morphogenesis and wiring.^88, 89^ Cadherin malfunction leads to information processing deficit noted in several neuropsychiatric disorders.^90^ Multiple genes from our data (PCDH15, PTPRU, WNT3, PVRL1, CDH12, DSG3, PRKCE, CDH13, USH1C and ACTG2) were involved in the cell adhesion process, of which PCDH15, CDH12 and CDH13 are candidate risk genes for psychiatric illness including SZ, PBP, ADHD and autism.^90^ N-cadherin subtype (CDH12) of cadherin family is regulated by DISC1 at cell membranes in primary neurons.^91^

We were unable to conduct replication analyses to confirm the current findings; however, several candidate genes and biological processes reported here are previously implicated in SZ and PBP, supporting a neurodevelopmental hypothesis of SZ and affective disorders.^92, 93^ The current findings are in agreement with prior pathway based studies that revealed similar mechanisms including axon guidance,^94, 95, 96^ cell adhesion^97, 98^ and glutamate pathways^93^ that were associated with psychosis. A recent BSNIP functional magnetic resonance imaging-genetic study^32^ using para-ICA identified several overlapping pathways and processes including axon guidance, developmental neurogenesis, and synaptic cell adhesion mediating functional abnormalities in psychosis. These data help validate the current approach undertaken to merge functional and genetic data to dissect the complex mechanisms mediating biological phenotypes in these disorders. A complete overlap in pathways and processes across diverse phenotypes is unlikely as they probably capture different neurophysiology.

### Medication effects

Several confounding factors including medication effects, illness chronicity, severity and duration may contribute to abnormal EEG activity noted in probands. Although prior studies report no medication influence on EEG frequency activity,^18^ it is likely that delta and theta activity might be driven by medications.^99, 100^ Delta activity implicated in this study was abnormal in relatives of SZ probands in an earlier related study;^19^ thus, it is unlikely that delta activity is influenced by antipsychotics, since relatives were not taking antipsychotic medications in that study. Although chlorpromazine equivalent dosage data were available for only subset of probands in our sample, no significant association was found with delta and theta activities. It was not feasible to account for medication effects in the present study, since probands were on several medications with varying dosages and durations. The study did not collect detailed longitudinal medication histories for probands to completely assess possible historical medication influence on eyes-open EEG frequency activity.

## Limitations

Advantages in the present study included use of dense spatial EEG data from a moderate size multisite sample and data-driven spatio-spectral characterization of EEG, which in turn was linked to genetic variants using multivariate association analysis. There were some limitations in this study. For genome-wide association study, the present sample is statistically underpowered, but such modest-sized sample can be dealt with statistically efficient multivariate association methods. Although the current sample comprised groups not matched on age, sex or across data collection sites, these effects were regressed through *post hoc* partial correlation between the LCs of EEG and SNP modalities. We emphasize caution on the interpretability of the present findings, as the current strategy for addressing the potential confounding effects from demographic factors can be verified only with a replication sample. However, prior studies have used similar strategies to address this issue.^30, 32^ The current study examined genetic association based on an additive model and was unable to capture epistatic genetic effects. Medication may be a confounding factor with EEG frequency activity. This issue will be addressed in future studies by including data from relatives (blood samples were collected) who were not taking antipsychotic medications. Relatives were not included in this study as the genotype data were unavailable. The para-ICA multivariate association model was unable to include all the genotyped SNPs owing to high-dimensional data issues. Therefore, other genetic networks may not be identified in the association. A replication sample was unavailable to confirm the current findings.

## Conclusions

Through this study, we identified several plausibly linearly interacting novel and known SZ and PBP susceptibility candidate genes mediating eyes-open EEG frequency abnormalities in both disorders using a multi-loci model based on para-ICA. We note that identified genes were overwhelmingly involved in plausible central nervous system function and pathways. Epistatic relationship between genes will be investigated in future studies. We identified delta and theta frequency abnormalities, associated with psychoses, related to two separate genetic networks each comprising gene groups enriched for developmental neurogenesis. Cell adhesion based on cadherin and synaptic contact was another key process associated with theta abnormalities in psychoses. Our data indicate a possible multi-loci genetic component associated with psychoses, encompassing individual genes playing crucial roles in brain development and function.

## Figures and Tables

**Table 1:** Demographic characteristics of study sample including probands and HCs

	*HC*	*SZ*	*PBP*	*Statistic*	P*-value*
Subjects (*N*)	56	105	145		
Mean (s.d.) age in years^a^	37.07 (11.28)	31.20 (11.08)	34.29 (12.05)	F(2,303)=4.99	*P*<0.007
Mean epochs (s.d.)	113.84 (33.03)	116.97 (35.63)	114.27 (37.79)	F(2,303)=0.21	0.8
Sex (male/female)^b^	22/34	79/26	60/85	*χ*^2^(2)=32.88	*P*<7.22e−8
Baltimore	11	36	41	—	—
Boston	3	5	3	—	—
Chicago	1	8	31	—	—
Dallas	0	1	5	—	—
Detroit	11	13	10	—	—
Hartford	30	42	55	—	—
Group-by-site effects	—	—	—	*χ*^2^(10)=31.95	*P*<4.07e−4
PANSS-positive mean (s.d.) range	—	15.09 (5.28) 7–31	14.04 (5.56) 7–38	—	—
PANSS-negative mean (s.d.) range	—	16.21 (5.73) 7–33	12.84 (4.68) 7–29	—	—
PANSS-general mean (s.d.) range	—	30.43 (8.73) 16–56	29.41 (8.51) 16–57	—	—
SBS Range Mean (s.d.)	—	7.52 (1.34) 4–9	2.09 (1.73) 0–6	—	—
CPZ equivalents^c^	—	542.01 (410.71)	383.31 (339.3)		
Schizoaffective disorder	0%	20%	25.5%		
Caucasian	30	57	95		
African-American	18	30	27		
Hispanic	2	10	17		
Asian	3	1	1		
Mixed	3	7	5		
Group-by-race effects	—	—	—	*χ*^2^(8)=14.77	*P*<0.063

Abbreviations: CPZ, chlorpromazine; HC, healthy controls; PANSS, positive and negative syndrome scale; PBP, psychotic bipolar disorder; SBS, schizo–bipolar scale; SZ, schizophrenia.

Effects of age, sex, race and data collection site were accounted for in the partial correlation assessing EEG-genetics associations.

a Healthy control group >schizophrenia probands.

b Disproportionate number of males in probands and disproportionate number of females in psychotic bipolar probands.

c CPZ equivalent dosage data were available for 61 schizophrenia and 82 psychotic bipolar probands.

**Table 2:** Pathways and gene information for top 20 unique genes from multivariate EEG-genetic association analysis.

*Gene networks*						
*Gene*	*SNP*	*CHR*	*Position*	*ZS*	*RW*	*Functional attribute/pathway involved*
*G1*						
MSRA^a^	rs11250004^I^	8p23.1	10 270 958	−5.78	1	Protection from oxidative damage
CD200^a^	rs12106673^I^	3q13.2	112 061 731	4.74	0.82	T-CP, regulates myeloid cell activity/IS
BLK^a^	rs2618451^I^	8p23.1	11 376 266	−4.31	0.74	CPD, stimulates insulin synthesis/IS, FA
TBC1D12^a^	rs1931757^I^	10q23.33	96 229 166	−4.27	0.73	GTPase-activating protein for Rab proteins
CLTCL1^a^	rs1061325^Ca^	22q11.21	19 184 095	4.1	0.709	Clathrin coated vesicles/SV, endocytosis
CYP2C19^a^	rs10786172^I^	10q23.33	96 581 094	−4.09	0.707	Catalyze reactions in drug metabolism/estrone, AA metabolism
CDK14^a^	rs732956^I^	7q21.13	90 589 751	4.07	0.704	Neuron differentiation and CNS development
DISC1^a^	rs9432040^I^	1q42.2	232 014 116	3.94	0.681	Neurite growth and cortical development
DDR2^a^	rs4656376^I^	1q23.3	162 640 649	3.81	0.659	CC, Regulation of CG and CD/adhesion, CS
SLC2A12	rs1385066^Utr-3^	6q23.2	134 310 874	−3.80	0.657	Catalyzes uptake of sugars/glucose transport
CACNG4	rs2108822^I^	17q24.2	65 021 998	3.75	0.648	Regulates AMPA glutamate receptors, synapse/CS, neuronal system and glutamate binding
CCDC88C	rs1951251^I^	14q32.11	91 859 263	−3.72	0.643	Regulates WNT signaling, protein phosphorylation
BICC1^a^	rs11815410^I^	10q21.1	60 516 335	3.71	0.641	Modulates protein translation during embryonic development, cadherin-based CA
TBCD^a^	rs8067926^I^	17q25.3	80 838 588	3.68	0.636	Tubulin folding, modulate microtubule dynamics/protein folding and metabolism
SGCZ	rs17119719^I^	8p22	14 392 321	−3.65	0.631	Involved in ECM, muscle cell development and homeostasis
C12ORF56^a^	rs10878163^I^	12q14.2	64 757 513	−3.64	0.629	Unknown
PLEKHG1^a^	rs6557088^I^	6q25.1	151 041 385	−3.62	0.626	Unknown
NARS2^a^	rs10501429^Utr-5^	11q14.1	78 279 790	3.61	0.624	Unknown/aminoacyl tRNA biosynthesis
TNKS	rs7840706^I^	8p23.1	9 535 056	−3.57	0.617	Regulates telomere length, vesicle trafficking/NAD metabolism
GAB2	rs2248407^C^	11q14.1	77 937 800	3.56	0.615	Regulates signaling pathways/NS, immune response Fc epsilon RI
						
*G3*						
CACNA1I^a^	rs3788568^I^	22q13.1	40 011 273	−4.66	1	Regulates neuronal excitability, signaling and ST/AG, CS and MAPK signaling
SLC44A5^a^	rs625357^I^	1p31.1	75 754 161	4.6	0.987	CP, choline transport/phospholipid metabolism
EPB41L4B	rs4978787^I^	9q31.3	112 024 496	−4.59	0.984	Axonal development
NTRK3	rs3784429^I^	15q25.3	88 610 457	−4.15	0.89	CD, proprioceptive neuron development/NS and apoptosis
SVIL^a^	rs1247089^I^	10p11.23	30 000 738	−4.14	0.888	Involved in cell spreading and disassembly of focal adhesions/androgen receptor activity
CLMP^a^	rs6589969^I^	11q24.1	123 013 271	4.08	0.875	Cell–cell adhesion
TBC1D12^a^	rs1931757^I^	10q23.33	96 229 166	−3.91	0.839	Refer to G1
REC8^a^	rs3736840^I^	14q12	24 647 522	−3.76	0.806	Involved in meiosis/meiotic synapsis
PID1^a^	rs6729811^I^	2q36.3	230 079 538	3.75	0.804	Proliferation of preadipocytes
TRPC4^a^	rs1415601^I^	13q13.3	38 440 568	−3.75	0.804	Regulates neurotransmitter release and CP/AG, CS and Netrin-1 signaling
DOCK8	rs10967788^I^	9p24.3	282 180	−3.75	0.804	Rho GTPases interactors
CSMD1^a^	rs2194604^I^	8p23.2	3 964 022	−3.69	0.791	CA, signal transduction/CAM and complement component
PSD3^a^	rs11785914^I^	8p22	18 698 031	3.69	0.791	Unknown/endocytosis
CD200^a^	rs12106673^I^	3q13.2	112 061 731	3.65	0.783	Refer to G1
CAPN9^a^	rs872505^I^	1q42.2	230 904 268	−3.56	0.763	Ca^2+^ and calmodulin binding
C16ORF73^a^	rs7200137^I^	16p13.3	1 901 211	3.56	0.763	Unknown
MSRA^a^	rs11250004^I^	8p23.1	10 270 958	−3.53	0.757	Refer to G1
UNC13C^a^	rs1124991^I^	15q21.3	54 384 302	−3.51	0.753	Synaptic vesicle exocytosis, regulate ST/SV
TRPM3	rs7860377^I^	9q21.12	73 312 129	−3.51	0.753	Regulates cellular CS, ion transport/intracellular CS
SLC02B1	rs2712818^I^	11q13.4	74 865 831	3.49	0.748	Regulates placental uptake of sulfated steroids/transport of organic anions and nucleosides

Abbreviations: AA, Arachidonic acid; AG, axon guidance; C, coding; CA, cell adhesion; CAM, cell adhesion molecule; CC, cell communication; CD, cell differentiation; CG, cell growth; CHR, chromosome; CNS; central nervous system; CP, cell proliferation; CPD, cell proliferation and differentiation; CS, calcium signaling; ECM, extracellular matrix; EEG, electroencephalogram; HC, histocompatibility complex; I, Intronic; IS, immune system; M, missense; MAPK, mitogen-activated protein kinases; NS, neurotrophin signaling; RW, relative weights (absolute *Z*-score divided by the maximum absolute SNP weight); SNP, single-nucleotide poymorphism; ST, synaptic transmission; SV, synaptic vesicle; Utr-3, three prime untranslated region; Utr-5, five prime untranslated region; ZS, *Z*-score.

a Multiple SNP occurrences (>2) of the gene within the genetic network.

## Notes

JAS has received support from Takeda, BMS, Lilly, Roche and Janssen. GR is the president of Genomas. MSK has received support from Sunovion. CAT has received funding from AStellas, Lilly, Iintracellular Therapies, Lundbeck and Pure Tech Ventures. All of these sources of support are unrelated to this study. The remaining authors declare no conflict of interest.
