# Supplementary material for: Multivariate genetic determinants of EEG oscillations in schizophrenia and psychotic bipolar disorder from the BSNIP study
Source: Transl Psychiatry. 2015 Jun 23;5(6):e588–. doi: 10.1038/tp.2015.76 (PMC4490286; doi:10.1038/tp.2015.76)
Supplement: Supplementary Information [file tp201576x1.doc]

Multivariate Genetic Determinants of EEG Oscillations in Schizophrenia and Psychotic Bipolar Disorder from the BSNIP Study

Supplementary Online Material

Materials and Methods

*Participant Recruitment*

Patients were recruited from inpatient and outpatient units at the six centers as part of the BSNIP study via community advertisements, online postings and local national association on mental illness. Participants completed Structured Clinical Interviews for the Diagnostic (SCID) and Statistical Manual of Mental disorders (DSM-IV-TR) diagnoses (SCID patient or nonpatient version as appropriate) reviewed and confirmed by a senior psychiatrist/psychologist and experienced clinicians. All probands were evaluated on the Positive and Negative Symptom Scale (PANSS)[^1^](#_ENREF_1), Young Mania Rating Scale[^2^](#_ENREF_2), Montgomery Asberg Depression Rating Scale (MADRS)[^3^](#_ENREF_3), and the Global Assessment of Functioning scale (GAF; axis V of DSM-IV). Probands were on stable doses of medication ≥ 4 weeks (Supplementary Table. S1), while healthy controls took no psychotropic medications.

*Eyes-Open EEG Data Acquisition*

Electrodes were positioned per the standard 10-10 system with forehead as ground and nose reference. Reference electrooculogram recordings were collected by placing one electrode at the inner and another at the outer canthus of the left and right eye. Subjects were instructed to sit quietly on a straight-backed chair in a shielded room with eyes open focused on a fixation cross on a monitor for 5 minutes. EEG data were digitized at sampling rate of 1000 Hz.

*EEG Data Processing*

Initial 10 sec of recording were discarded. Faulty electrodes were inspected and corrected (< 8% for any subject) using spline interpolation. Blink and cardiac artifacts were removed by using independent component analysis. Epochs were constructed from continuous data by segmenting into 50% overlapping packets of 2.048 sec. Individual epochs were baseline-corrected using mean voltage activity. Epochs were safeguarded from extreme outliers (> 150uV threshold) and artifact rejected using improbable distribution (> 3.25 standard deviation from mean), and kurtotic behavior (3.75 standard deviation from mean). Epochs were subjected to spectral-transformation using a Hamming window and those exceeding (+/-) 4 standard deviations from the mean spectral amplitude at all frequency points between 0.5-50 Hz were excluded.

*Frequency Transformation and Data Reduction using Group Independent Component Analysis (GICA)*

Instantaneous EEG frequency-amplitude profile at 64 electrodes from both probands (schizophrenia + psychotic bipolar disorder), their relatives and controls were used in GICA to identify spatial maps associated with independent spectral networks representing various neural substrates[^4^](#_ENREF_4). Each subject’s data was organized (see Supplementary Figure S1 and also refer to[^4^](#_ENREF_4) for detailed description) by concatenating the amplitude spectral profile for all spatial leads across the epoch dimension. Missing epochs were imputed with the mean spectral data across valid epochs. A simple data reduction procedure using Principal Component Analysis (PCA) at the subject level was employed to reduce the spatial dimension, followed by spatial compounding of spectral data from all subjects. A second data reduction step was applied to compress the spatially and spectrally concatenated data. The number of independent components for the EEG frequency data was selected as 8 using minimum description length criteria [^5^](#_ENREF_5) and ICASSO[^6^](#_ENREF_6), a consistency check tool within EEGIFT[^7^](#_ENREF_7) to avoid overfitting.

GICA extracted spectral series and a spatial map (representing brain regions or electrodes comprising each frequency network) for each component, based on the overall group characteristics. The EEG spectral series and spatial maps were then back-reconstructed for each subject producing a series of spatial maps and component spectral profile capturing individual differences in the ICA components derived from the variations common to all subjects. The spatial weights reflect the contribution of the individual leads to the associated frequency activity. To gain statistical efficiency, the pooled spectral data from probands, relatives and controls were used in GICA, however only data specific to subset of subjects (N=306) used in this study were selected from the GICA reduced data for the final multivariate association analysis. Spatial topographic coefficients for all the 8 frequency components were concatenated as a single vector (8 x 64=512) to serve as the EEG oscillatory phenotype for each subject.

*Advantages of SNP-EEG Parallel ICA (Para-ICA) Association*

The multivariate Para-ICA model offers the capability to include multiple gene variants and several complex phenotypes compared to a single biomarker in univariate analysis. A single biomarker may lack the power to drive an association due to its incomplete representation and thus a comprehensive biomarker subset would enhance the association power and predictive efficacy. The main advantages of Para-ICA method are: 1) data-driven analysis with no assumption on the data distribution, 2) unsystematic complex noise source isolation from relevant biological signals yielding better signal-to-noise ratio 3) managing large scale data from multiple data sources by data fusion and reduction, thus improving statistical predictive efficacy by accounting for multiple comparisons on reduced factors.

Results

*Eyes-Open EEG Frequency Components from GICA*

Two delta (1.5 – 4 Hz) components (N6 & N8 see Supplementary Figure S3) were identified with a peak at 1.5 Hz and 4 Hz respectively with a topographic distribution localized to the anterior and posterior regions respectively. One theta component N1 with a peak between 4.15-8 Hz was noticed with a frontal to posterior and central distribution respectively. Slow alpha (N3) and fast alpha components (N2 and N5) were validated by a peak between 8.15-10 Hz and 10.15-13 Hz respectively with maximum amplitude distribution over central-parietal and parietal regions. The slow beta component (N4) had a peak within 13.15-20 Hz range with a central/parietal distribution. The fast beta component (N7) was validated by the peak at 25 Hz and having a maximal distribution localized frontally.

**Supplementary Figure S1:** Processing pipeline for eyes-open EEG data and quality control (QC) steps for single nucleotide polymorphism (SNP) data.

EEG data were artifact rejected and compressed to P=8 frequency components and associated 64 spatial weights using group independent component analysis (GICA).

The pruning process for SNP data involved two stages: individual based followed by SNP marker based QC. Poorly genotyped individuals (n=17) and bad SNPs were removed from the final regression analysis. The SNP sample (M) retained after each QC step is shown in the figure. A total of N=603 subjects and M=575,689 SNPs were retained after the quality control. SNP data were corrected for population stratification bias by correcting for top 3 Eigen factors associated with self-reported ethnicity. SNPs were further reduced to 10,422 markers using logistic regression. The final data (N=306 subjects and M=10,422 SNPs) for multivariate analysis was extracted from the pruned data set.

LD, linkage disequilibrium; ICA, Independent Component Analysis

**
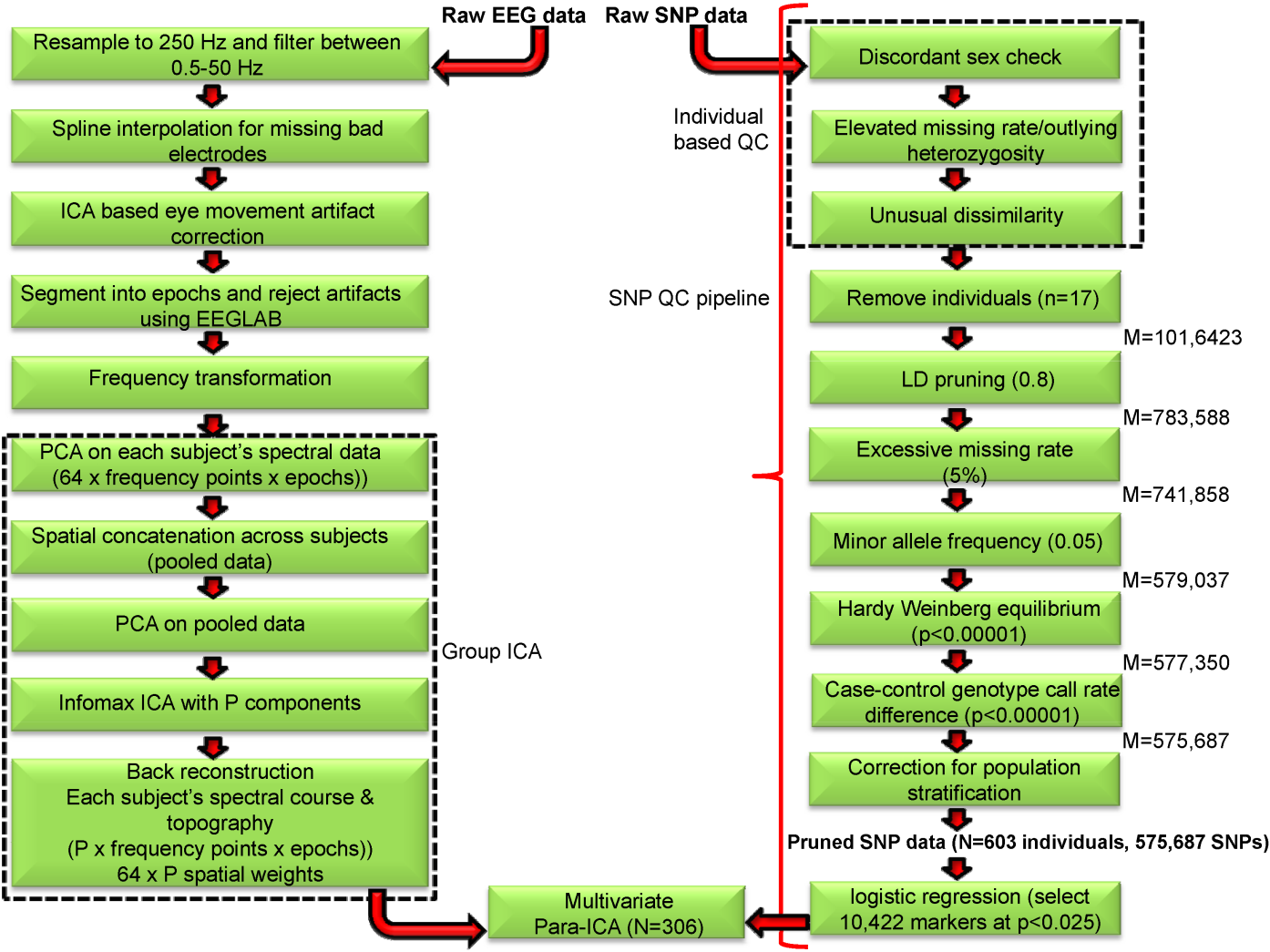
**

**Supplementary Figure S2:** Schematic illustration of the parallel independent component analysis (Para-ICA) for genetic association of spatial EEG frequency activity.

Para-ICA extracts components or hidden sources from spatio-spectral EEG and SNP data by jointly maximizing the independence between components in each modality and optimizing the inter-modality correlation. Data were constructed as a matrix of subjects by SNP (K=306 X M=10,422) and subjects by spatial weights (K=306 X P= 512 (8 x 64) spatial weights)) associated with the frequency components. The number of sources extracted from Para-ICA for the EEG and SNP data were N_1_ = 5 and N_2_ = 9 respectively.

LC, loading coefficient; SNP, single nucleotide polymorphism

**
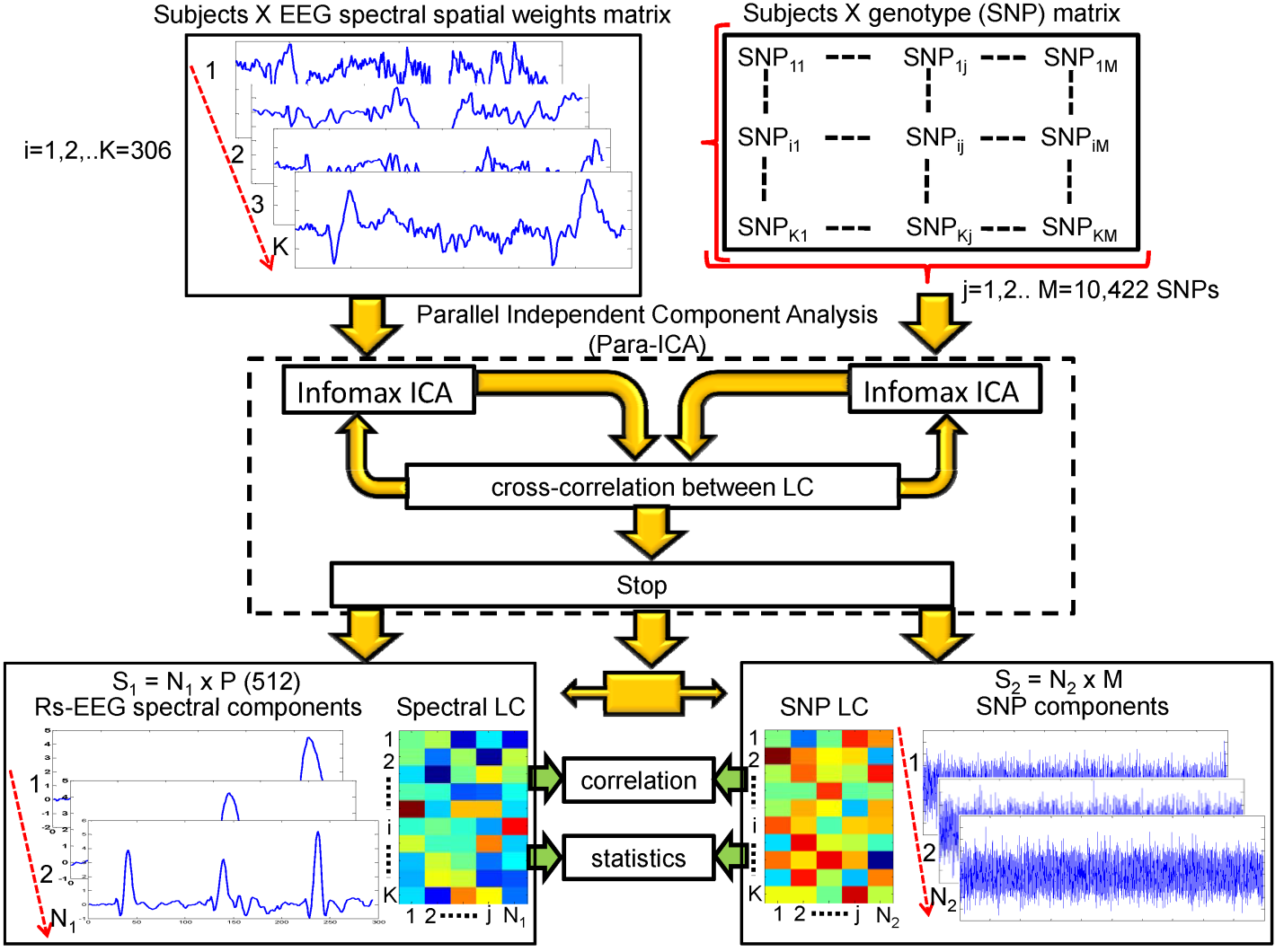
**

**Supplementary Figure S3:** Data-driven eyes-open EEG frequency components and associated topography from group independent component analysis (GICA).

The frequency components were averaged across epochs and all subjects (N=1271, including probands, relative and controls). The components are randomly ordered in GICA but we have reordered the eight spectral components from low to high frequency (1.5-50 Hz). The spatial weights of each frequency component are dimensionless measure representing the strength of the connection between each electrode and that component. The spatial weights were tested for outliers and N=180 subjects were excluded. Thus, N=1091 subjects were used for assessing group differences, of which N=306 subjects had genotyping data and were used in the current study to evaluate multivariate genetic association.

**
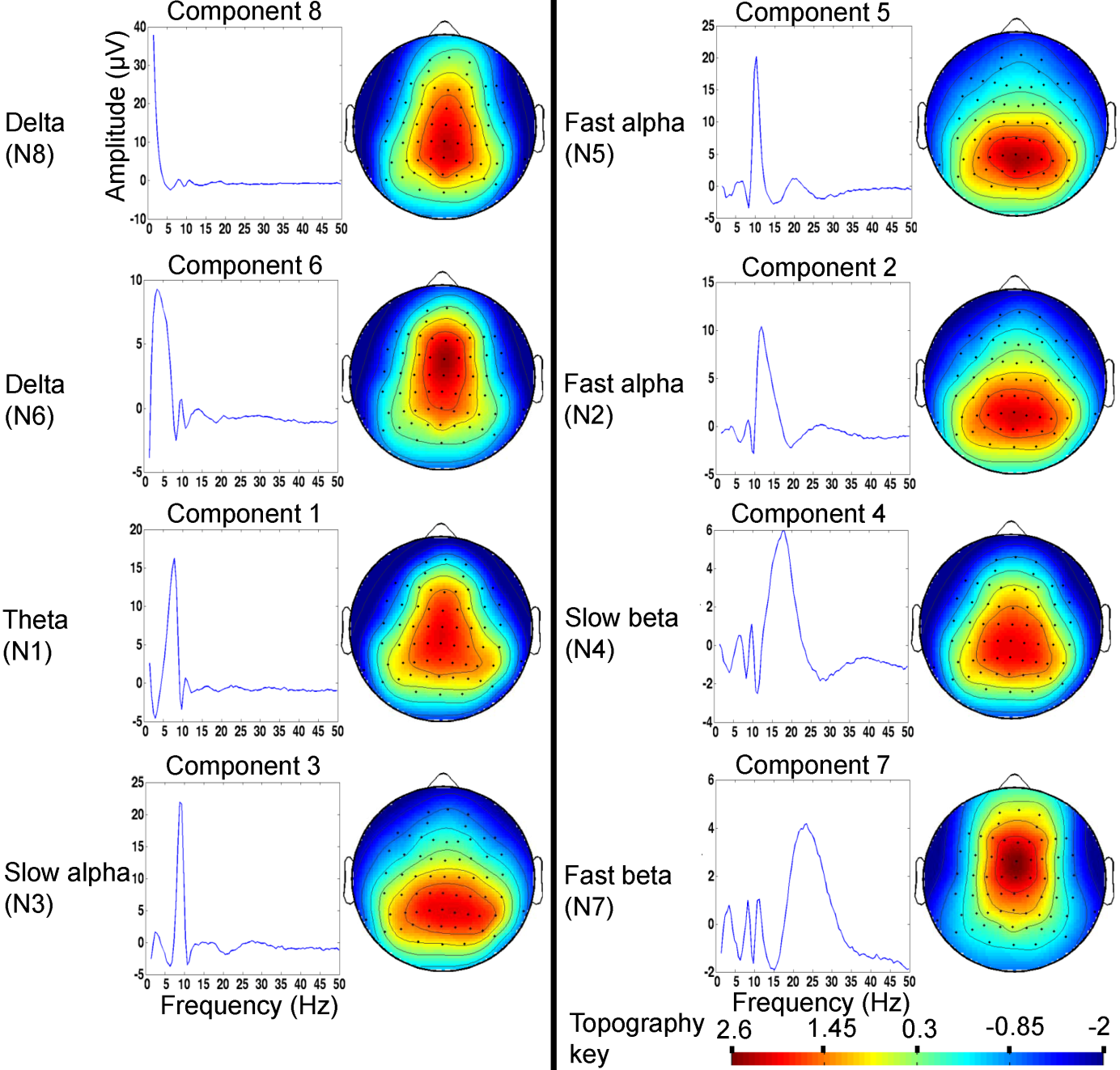
**

**Supplementary Table S1:** Medication information for proband and control subjects.

|  | HC (n=56) | SZ (n=105) | PBP (n=145) |
| --- | --- | --- | --- |
|  |  |  |  |
| Unknown medication history, % (n) | 48.2 (27) | 3.85 (4) | 4.13 (6) |
|  |  |  |  |
| Medication data below are for subjects with medication history reported | 29 | 101 | 139 |
| Not on psychotropic medications, % (n) | 93.10 (27) | 0.9 (1) | 0.68 (1) |
|  |  |  |  |
| Anticholinergic/Antiparkinsonian, % (n) | 0 (0) | 17.82 (18) | 10.34 (15) |
| Antidepressant (Any), % (n) |  |  |  |
| A. Tricyclic | 0 (0) | 9.9 (10) | 16.5 (24) |
| B. MAO inhibitors | 0 (0) | 0 (0) | 0 (0) |
| C. SSRI/SNRI | 3.4 (1) | 32.67 (33) | 24.82 (36) |
| D. Miscellaneous | 0 (0) | 6.93 (7) | 15.17 (22) |
| Antipsychotic (Any), % (n) |  |  |  |
| A. First generation | 0 (0) | 26.7 (27) | 11.72 (17) |
| B. Second generation | 0 (0) | 92.07 (93) | 70.3 (102) |
| Anxiolytic/Hypnotic, % (n) | 3.4 (1) | 20.79 (21) | 26.89 (39) |
| Mood Stabilizer (Any), % (n) |  |  |  |
| A. Lithium | 0 (0) | 5.94 (6) | 27.58 (40) |
| B. Anticonvulsants | 0 (0) | 27.72 (28) | 59.3 (86) |
| Miscellaneous, Centrally Active, % (n) | 0 (0) | 15.84 (16) | 16.5 (24) |
| Stimulants, % (n) | 0 (0) | 4.95 (5) | 9.6 (14) |

HC, healthy controls; MAO, monoamine oxidase inhibitor; PBP, psychotic bipolar disorder; SNRI, serotonin-norepinephrine reuptake inhibitors; SSRI, selective serotonin reuptake inhibitors; SZ, schizophrenia

**Supplementary Table S2:** Results of enrichment analysis including GeneGo pathway maps, process networks, metabolic networks and gene ontology processes associated with contributing genes from network G1 and G3. Bold P-values indicate significant (p<0.05) after FDR correction.

| **Pathways for G1** | | | |
| --- | --- | --- | --- |
| **Pathway maps** | P  (uncorrected) | p  (FDR) | Ratio = In data/Total |
| wtCFTR and delta508 traffic/clathrin coated vesicles formation | 1.17E-3 | 0.19 | 3/19 |
| Development GDNF family signaling | 1.72E-3 | 0.19 | 4/46 |
| Neurophysiological process NMDA-dependent postsynaptic long term potentiation | 2.02E-3 | 0.19 | 5/80 |
| Development EDNRB signaling | 2.34E-3 | 0.19 | 4/50 |
| Muscle contraction ACM regulation of smooth muscle contraction | 3.55E-3 | 0.21 | 4/56 |
| Development slit-robo signaling | 4.51E-3 | 0.21 | 3/30 |
| DNA damage role of Brca1 and Brca2 in DNA repair | 4.51E-3 | 0.21 | 3/30 |
| Beta-2-adrenergic-dependent CFTR expression | 1.19E-3 | 0.4 | 2/15 |
| G-protein signaling regulation of cAMP levels by ACM | 1.39E-2 | 0.4 | 3/45 |
| Transcription factor tubby signaling pathways | 1.53E-2 | 0.4 | 2/17 |
| **Process networks** |  |  |  |
| Development_neurogenesis_synaptogenesis | 2.43E-5 | **3.18E-3** | 14/180 |
| Cell adhesion_cadherins | 1.01E-4 | **5.62E-3** | 13/180 |
| Cell adhesion_synaptic contact | 1.28E-4 | **5.62E-3** | 13/184 |
| Cell adhesion cell-matrix interactions | 4.95E-3 | 0.13 | 11/211 |
| Cytoskeleton_regulation of cytoskeleton rearrangement | 5.26E-3 | 0.13 | 10/183 |
| Cell adhesion_amyloid proteins | 8.14E-3 | 0.16 | 10/195 |
| Apoptosis_Anti-apoptosis mediated by external signals via PI3K/AKT | 1.01E-2 | 0.16 | 11/233 |
| Cytoskeleton_cytoplasmic microtubules | 1.07E-2 | 0.16 | 7/115 |
| Development_skeletal muscle development | 1.10E-2 | 0.16 | 8/144 |
| Neurophysiological process_Long-term potentiation | 2.93E-2 | 0.35 | 5/82 |
| **Metabolic networks** |  |  |  |
| Glutamic acid pathways | 7.37E-5 | **1.05E-2** | 6/103 |
| Glutamic acid transport | 4.8E-4 | **3.28E-2** | 6/145 |
| 6’-sialyllactose pathways and transport | 6.89E-4 | **3.28E-2** | 5/102 |
| L-glutamate pathways and transport | 2.11E-3 | **7.56E-2** | 5/131 |
| Maltohexaose pathways and transport | 1.24E-2 | 0.32 | 4/129 |
| Maltopentaose pathways and transport | 1.41E-2 | 0.32 | 4/134 |
| L-arginine pathways and transport | 1.83E-2 | 0.32 | 3/80 |
| Lyso-Phosphatidylserine pathway | 1.89E-2 | 0.32 | 3/81 |
| CYP3A4-11-Glucagon-HNF4 | 2.57E-2 | 0.32 | 3/91 |
| Acyl-L-carnitine pathways | 2.65E-2 | 0.32 | 3/92 |
| **Go processes** |  |  |  |
| Axon guidance | 5.13E-11 | **1.06E-7** | 32/490 |
| Calcium ion transmembrane transport | 6.58E-10 | **6.81E-7** | 17/145 |
| Cell adhesion | 1.38E-9 | **9.51E-7** | 41/981 |
| Synaptic transmission | 2.75E-9 | **1.42E-6** | 34/825 |
| Axongenesis | 8.81E-9 | **3.64E-6** | 16/675 |
| Transmembrane receptor protein tyrosine kinase signaling pathway | 2.06E-8 | **7.13E-6** | 14/706 |
| Intracellular signal transduction | 1.27E-7 | **3.75E-5** | 25/1778 |
| Oocyte maturation | 2.13E-7 | **5.52E-5** | 7/24 |
| Renal inner medulla development | 6.18E-7 | **1.42E-4** | 4/5 |
| cAMP catabolic process | 1.35E-6 | **2.55E-4** | 6/20 |
| **Pathways for G3** | | | |
| **Pathway maps** |  |  |  |
| Development_role of CDK5 neuronal development | 7.33E-4 | 0.14 | 4/34 |
| G-protein signaling H-RAS regulation pathways | 1.01E-3 | 0.14 | 4/37 |
| Transcription receptor mediated HIF regulation | 1.24E-3 | 0.14 | 4/39 |
| Role of alpha-6/beta-4 integrins in carcinoma progression | 2.12E-3 | 0.17 | 4/45 |
| Development PIP3 signaling in cardiac myocytes | 2.49E-3 | 0.17 | 4/47 |
| Cell adhesion ECM remodeling | 3.62E-3 | 0.21 | 4/52 |
| Apoptosis and survival role of CDK5 in neuronal death survival | 8.03E-3 | 0.35 | 3/34 |
| G-protein signaling regulation of RAC1 activity | 9.42E-3 | 0.35 | 3/36 |
| Development MAG-dependent inhibition of neutrite outgrowth | 1.01E-2 | 0.35 | 3/37 |
| Cell cycle nucleocytoplasmic transport of CDK/Cyclins | 1.22E-2 | 0.35 | 2/14 |
| **Process networks** |  |  |  |
| Development neurogenesis synaptogenesis | 1.18E-4 | **0.017** | 13/180 |
| Cell adhesion synaptic contact | 1.9E-3 | 0.13 | 11/184 |
| Development neurogenesis axonal guidance | 3.73E-3 | 0.16 | 12/230 |
| Cytoskeleton actin filaments | 4.39E-3 | 0.16 | 10/176 |
| Apoptosis Anti-Apoptosis mediated by external signals via PI3/AKT | 1.11E-2 | 0.32 | 11/233 |
| Cell adhesion cell matrix interactions | 1.49E-2 | 0.36 | 10/211 |
| Regulation of metabolism regulation of lipid metabolism | 1.76E-2 | 0.36 | 5/71 |
| Cell adhesion attractive and repulsive attractors | 3.39E-2 | 0.4 | 8/175 |
| Development skeletal muscle development | 3.46E-2 | 0.4 | 7/144 |
| Proteolysis ECM remodeling | 3.52E-2 | 0.4 | 5/85 |
| **Metabolic networks** |  |  |  |
| Phosphatidylnositols-4,5-diphosphate pathways | 1.02E-3 | 0.08 | 5/92 |
| N-acyl-sphingosine phosphate pathways | 1.41E-3 | 0.08 | 5/99 |
| 6’-sialyllactose pathways and transport | 1.62E-3 | 0.08 | 5/102 |
| CYP3A4-3-insulin-C/EBP-IRS2 | 2.26E-3 | 0.09 | 5/110 |
| Lyso-Phsophatidylserine pathway | 4.74E-3 | 0.15 | 4/81 |
| CYP3A4-3-insulin-C/EBP-IRS1 | 8.62E-3 | 0.18 | 4/96 |
| CYP2C9-3-Glucagon-HNF4alpha | 9.59E-3 | 0.18 | 4/99 |
| CYP3A4-1-Insulin-C/EBP-IRS2 | 9.93E-3 | 0.18 | 4/100 |
| CYP3A4-4-Insulin-C/EBP-IRS2 | 1.1E-2 | 0.18 | 4/103 |
| Glutamic acid pathway | 1.1E-2 | 0.18 | 4/103 |
| **GO processes** |  |  |  |
| Transmembrane receptor protein tyrosine kinase signaling pathways | 2.31E-14 | **4.76E-11** | 20/706 |
| Axon guidance | 1.26E-9 | **1.3E-6** | 30/490 |
| Cell adhesion | 5.72E-8 | **3.92E-5** | 38/981 |
| Nervous system development | 1.8E-7 | **9.27E-5** | 32/2500 |
| Protein localization to synapse | 1.44E-6 | **5.92E-4** | 6/21 |
| Regulation of neurotransmitter secretion | 2.43E-6 | **8.23E-4** | 7/76 |
| Glomerular filtration | 2.8E-6 | **8.23E-4** | 5/13 |
| Principal sensory nucleus of trigeminal nerve development | 6.9E-6 | **1.59E-3** | 3/3 |
| Synapse assembly | 7.15E-6 | **1.59E-3** | 9/78 |
| Gilal cell differentiation | 7.76E-6 | **1.59E-3** | 6/196 |

ACM, adipocyte-conditioned medium; cAMP, Cyclic adenosine monophosphate; CDK, cyclin-dependent kinase; CFTR, cystic fibrosis transmembrane regulator; EBP, enhancer binding proteins; IRS, insulin receptor substrate; ECM, extracellular matrix; EDNRB, endothelin receptor type B; FDR, fasle discovery rate; GDNF, gilal cell line-derived neurotrophic factor (GDNF); HIF-1, hypoxia inducible factor-1; HNF4, hepatocyte nuclear factor 4; NMDA N-methyl-D-aspartate; PI3K, phosphatidylinositol-3-kinases, PIP3, Phosphatidylinositol (3,4,5)-triphosphate;

**Supplementary Table S3:** Brain regions and mental disorders associated with top 20 most significant genes from gene networks G1 and G3.

Expression Z-scores from Allen brain atlas database was used to identify brain regions, where the genes from G1 and G3 were expressed. The brain regions were selected based on association with EEG delta and theta activity from prior EEG-fMRI studies. Other mental disorders related to these genes from prior studies are also provided.

| G1 | Disease Risk | MFG | SFG | IFG | PHG | PCL | INS | PRC | MTG | IPL | STG | ACC | HIP |
| --- | --- | --- | --- | --- | --- | --- | --- | --- | --- | --- | --- | --- | --- |
| MSRA | AD[^8^](#_ENREF_8) & SZ[^9^](#_ENREF_9) |  | x | x | x | x | x |  |  |  | x | x | x |
| CD200 | PD[^10^](#_ENREF_10) |  |  | x | x |  | x |  | x | x | x |  | x |
| BLK |  | x | x | x |  | x | x | x | x | x | x | x | x |
| TBC1D12 |  |  |  |  |  |  |  |  |  |  |  |  |  |
| CLTCL1 |  |  |  |  |  |  |  |  |  |  | x |  |  |
| CYP2C19 | MDD[^11^](#_ENREF_11) | x | x | x | x |  | x |  | x |  | x | x | x |
| CDK14 |  |  |  | x |  | x | x | x |  | x | x |  | x |
| DISC1 | ASD[^12^](#_ENREF_12), MDD, PBP & SZ[^13^](#_ENREF_13) | x |  | x | x | x | x | x |  | x |  | x | x |
| DDR2 |  |  |  |  |  |  |  |  |  |  |  |  | x |
| SLC2A12 |  |  | x | x | x |  | x |  |  |  |  | x |  |
| CACNG4 |  |  |  |  |  | x |  |  |  |  |  |  | x |
| CCDC88C |  | x |  | x |  |  | x |  |  | x | x | x | x |
| BICC1 | D[^14^](#_ENREF_14) | x | x | x | x | x | x | x | x | x | x | x | x |
| TBCD |  | x | x | x | x | x | x | x | x | x | x | x | x |
| SGCZ |  |  |  | x |  | x | x | x |  | x | x | x | x |
| C12ORF56 |  |  |  | x |  | x | x | x |  | x | x | x | x |
| PLEKHG1 | PAD[^15^](#_ENREF_15) |  |  |  |  |  |  |  |  |  |  |  |  |
| NARS2 |  |  |  | x | x |  |  |  |  | x | x | x | x |
| TNKS |  |  |  |  |  |  |  |  |  |  |  |  | x |
| GAB2 | AD[^16^](#_ENREF_16) |  | x |  |  |  |  |  | x |  |  |  |  |
| G3 |  |  |  |  |  |  |  |  |  |  |  |  |  |
| CACNA1I |  | x | x | x | x |  | x | x |  | x | x | x |  |
| SLC44A5 |  |  |  | x | x |  | x | x | x | x | x |  |  |
| EPB41L4B |  | x | x | x | x |  | x | x | x | x | x | x |  |
| NTRK3 | D[^17^](#_ENREF_17), MDD[^18^](#_ENREF_18), PBP[^19^](#_ENREF_19) & SZ[^20^](#_ENREF_20) | x | x | x | x |  | x | x | x | x | x | x |  |
| SVIL |  | x |  | x | x |  | x | x | x | x | x | x |  |
| CLMP |  |  |  |  |  |  |  |  |  |  |  |  |  |
| TBC1D12 |  |  |  |  |  |  |  |  |  |  |  |  |  |
| REC8 |  |  |  |  |  |  |  |  |  |  |  |  |  |
| PID1 |  |  | x | x | x |  | x |  | x | x | x | x |  |
| TRPC4 |  |  |  |  | x |  | x |  |  |  | x | x |  |
| DOCK8 | MR[^21^](#_ENREF_21) |  |  | x |  |  |  |  |  |  | x | x |  |
| CSMD1 | SZ[^22^](#_ENREF_22) | x | x | x | x |  | x | x | x | x | x | x |  |
| PSD3 |  |  |  | x | x |  | x |  |  | x |  |  |  |
| CD200 | see G1 |  |  |  |  |  |  |  |  |  |  |  |  |
| CAPN9 |  | x | x | x | x |  | x |  | x | x | x | x |  |
| C16ORF73 |  | x |  | x | x |  | x |  | x | x |  |  |  |
| MSRA | See G1 |  |  |  |  |  |  |  |  |  |  |  |  |
| UNC13C |  |  |  |  |  |  |  |  |  |  |  |  |  |
| TRPM3 |  |  |  |  |  |  |  |  |  |  | x | x |  |
| SLC02B1 |  |  |  | x | x |  | x |  |  |  | x | x |  |

ACC, anterior cingulate; Alzheimer’s disease; ASD, autism spectrum disorder; D, depression; HIP, hippocampus; IFG, inferior frontal gyrus; INS, insula; IPL, inferior parietal lobule; ITG, inferior temporal gyrus; MDD, major depressive disorder; MFG, middle frontal gyrus, MTG, medial frontal gyrus; MR, mental retardation; PAD, panic disorder; PBP, psychotic bipolar disorder; PCL, paracentral lobule; PD, parkinson disease; PHG, parahippocampal gyrus; PRC, precuneous; SFG, superior frontal gyrus; STG, superior temporal gyrus; SZ, schizophrenia

**Supplementary References**

1. Kay SR. Positive-negative symptom assessment in schizophrenia: psychometric issues and scale comparison. *Psychiatr Q* 1990; **61**(3)**:** 163-178.

2. Young RC, Biggs JT, Ziegler VE, Meyer DA. A rating scale for mania: reliability, validity and sensitivity. *Br J Psychiatry* 1978; **133:** 429-435.

3. Montgomery SA, Asberg M. A new depression scale designed to be sensitive to change. *Br J Psychiatry* 1979; **134:** 382-389.

4. Narayanan B, O'Neil K, Berwise C, Stevens MC, Calhoun VD, Clementz BA*, et al*. Resting state electroencephalogram oscillatory abnormalities in schizophrenia and psychotic Bipolar Patients and their relatives from the bipolar and schizophrenia network on intermediate phenotypes study. *Biol Psychiatry* 2013.

5. Li YO, Adali T, Calhoun VD. Estimating the number of independent components for functional magnetic resonance imaging data. *Hum Brain Mapp* 2007; **28**(11)**:** 1251-1266.

6. Himberg J, Hyvarinen A, Esposito F. Validating the independent components of neuroimaging time series via clustering and visualization. *Neuroimage* 2004; **22**(3)**:** 1214-1222.

7. Eichele T, Rachakonda S, Brakedal B, Eikeland R, Calhoun VD. EEGIFT: group independent component analysis for event-related EEG data. *Comput Intell Neurosci* 2011; **2011:** 129365.

8. Gabbita SP, Aksenov MY, Lovell MA, Markesbery WR. Decrease in peptide methionine sulfoxide reductase in Alzheimer's disease brain. *J Neurochem* 1999; **73**(4)**:** 1660-1666.

9. Walss-Bass C, Soto-Bernardini MC, Johnson-Pais T, Leach RJ, Ontiveros A, Nicolini H*, et al*. Methionine sulfoxide reductase: a novel schizophrenia candidate gene. *Am J Med Genet B Neuropsychiatr Genet* 2009; **150B**(2)**:** 219-225.

10. Wang XJ, Ye M, Zhang YH, Chen SD. CD200-CD200R regulation of microglia activation in the pathogenesis of Parkinson's disease. *J Neuroimmune Pharmacol* 2007; **2**(3)**:** 259-264.

11. Sim SC, Nordin L, Andersson TM, Virding S, Olsson M, Pedersen NL*, et al*. Association between CYP2C19 polymorphism and depressive symptoms. *Am J Med Genet B Neuropsychiatr Genet* 2010; **153B**(6)**:** 1160-1166.

12. Kilpinen H, Ylisaukko-Oja T, Hennah W, Palo OM, Varilo T, Vanhala R*, et al*. Association of DISC1 with autism and Asperger syndrome. *Mol Psychiatry* 2008; **13**(2)**:** 187-196.

13. Hennah W, Thomson P, McQuillin A, Bass N, Loukola A, Anjorin A*, et al*. DISC1 association, heterogeneity and interplay in schizophrenia and bipolar disorder. *Mol Psychiatry* 2009; **14**(9)**:** 865-873.

14. Lewis CM, Ng MY, Butler AW, Cohen-Woods S, Uher R, Pirlo K*, et al*. Genome-wide association study of major recurrent depression in the U.K. population. *Am J Psychiatry* 2010; **167**(8)**:** 949-957.

15. Otowa T, Yoshida E, Sugaya N, Yasuda S, Nishimura Y, Inoue K*, et al*. Genome-wide association study of panic disorder in the Japanese population. *J Hum Genet* 2009; **54**(2)**:** 122-126.

16. Schjeide BM, Hooli B, Parkinson M, Hogan MF, DiVito J, Mullin K*, et al*. GAB2 as an Alzheimer disease susceptibility gene: follow-up of genomewide association results. *Arch Neurol* 2009; **66**(2)**:** 250-254.

17. Feng Y, Vetro A, Kiss E, Kapornai K, Daroczi G, Mayer L*, et al*. Association of the neurotrophic tyrosine kinase receptor 3 (NTRK3) gene and childhood-onset mood disorders. *Am J Psychiatry* 2008; **165**(5)**:** 610-616.

18. Verma R, Holmans P, Knowles JA, Grover D, Evgrafov OV, Crowe RR*, et al*. Linkage disequilibrium mapping of a chromosome 15q25-26 major depression linkage region and sequencing of NTRK3. *Biol Psychiatry* 2008; **63**(12)**:** 1185-1189.

19. Athanasiu L, Mattingsdal M, Melle I, Inderhaug E, Lien T, Agartz I*, et al*. Intron 12 in NTRK3 is associated with bipolar disorder. *Psychiatry Res* 2010; **185**(3)**:** 358-362.

20. Otnaess MK, Djurovic S, Rimol LM, Kulle B, Kahler AK, Jonsson EG*, et al*. Evidence for a possible association of neurotrophin receptor (NTRK-3) gene polymorphisms with hippocampal function and schizophrenia. *Neurobiol Dis* 2009; **34**(3)**:** 518-524.

21. Griggs BL, Ladd S, Saul RA, DuPont BR, Srivastava AK. Dedicator of cytokinesis 8 is disrupted in two patients with mental retardation and developmental disabilities. *Genomics* 2008; **91**(2)**:** 195-202.

22. Havik B, Le Hellard S, Rietschel M, Lybaek H, Djurovic S, Mattheisen M*, et al*. The complement control-related genes CSMD1 and CSMD2 associate to schizophrenia. *Biol Psychiatry* 2011; **70**(1)**:** 35-42.
